# Feasibility of neurally adjusted positive end-expiratory pressure in rabbits with early experimental lung injury

**DOI:** 10.1186/s12871-015-0103-z

**Published:** 2015-09-14

**Authors:** Ling Liu, Daijiro Takahashi, Haibo Qui, Arthur S. Slutsky, Christer Sinderby, Jennifer Beck

**Affiliations:** 1 Department of Critical Care Medicine, Nanjing Zhong-Da Hospital, Southeast University School of Medicine, 87 Dingjiaqiao Street, Nanjing, 210009 China; 2 Division of Pediatrics, Fukuda Hospital, 2-2-6, Shinmachi, Chuou-ku Kumamoto city, 860-0004 Japan; 3 Keenan Research Centre for Biomedical Science of St. Michael’s Hospital; Department of Critical Care, St. Michael’s Hospital, 30 Bond Street, Toronto, ON Canada M5B1W8; 4 Department of Medicine and Interdepartmental Division of Critical Care Medicine, University of Toronto, Toronto, Canada; 5 Department of Pediatrics, University of Toronto, Toronto, Canada; 6 Institute for Biomedical Engineering and Science Technology (iBEST) at Ryerson University and St-Michael’s Hospital, Toronto, Canada

## Abstract

**Background:**

During conventional Neurally Adjusted Ventilatory Assist (NAVA), the electrical activity of the diaphragm (EAdi) is used for triggering and cycling-off inspiratory assist, with a fixed PEEP (so called “Triggered Neurally Adjusted Ventilatory Assist” or “tNAVA”). However, significant post-inspiratory activity of the diaphragm can occur, believed to play a role in maintaining end-expiratory lung volume. Adjusting pressure continuously, in proportion to both inspiratory and expiratory EAdi (Continuous NAVA, or cNAVA), would not only offer inspiratory assist for tidal breathing, but also may aid in delivering a “neurally adjusted PEEP”, and more specific breath-by-breath unloading.

**Methods:**

Nine adult New Zealand white rabbits were ventilated during independent conditions of: resistive loading (RES^1^ or RES^2^), CO_2_ load (CO_2_) and acute lung injury (ALI), either via tracheotomy (INV) or non-invasively (NIV). There were a total of six conditions, applied in a non-randomized fashion: INV-RES^1^, INV-CO_2_, NIV-CO_2_, NIV-RES^2^, NIV-ALI, INV-ALI. For each condition, tNAVA was applied first (3 min), followed by 3 min of cNAVA. This comparison was repeated 3 times (repeated cross-over design). The NAVA level was always the same for both modes, but was newly titrated for each condition. PEEP was manually set to zero during tNAVA. During cNAVA, the assist during expiration was proportional to the EAdi. During all runs and conditions, ventilator-delivered pressure (Pvent), esophageal pressure (Pes), and diaphragm electrical activity (EAdi) were measured continuously. The tracings were analyzed breath-by-breath to obtain peak inspiratory and mean expiratory values.

**Results:**

For the same peak Pvent, the distribution of inspiratory and expiratory pressure differed between tNAVA and cNAVA. For each condition, the mean expiratory Pvent was always higher (for all conditions 4.0 ± 1.1 vs. 1.1 ± 0.5 cmH_2_O, *P* < 0.01) in cNAVA than in tNAVA. Relative to tNAVA, mean inspiratory EAdi was reduced on average (for all conditions) by 19 % (range 14 %–25 %), *p* < 0.05. Mean expiratory EAdi was also lower during cNAVA (during INV-RES^1^, INV-CO_2_, INV-ALI, NIV-CO_2_ and NIV-ALI respectively, *P* < 0.05). The inspiratory Pes was reduced during cNAVA all 6 conditions (*p* < 0.05). Unlike tNAVA, during cNAVA the expiratory pressure was comparable with that predicted mathematically (mean difference of 0.2 ± 0.8 cmH_2_O).

**Conclusion:**

Continuous NAVA was able to apply neurally adjusted PEEP, which led to a reduction in inspiratory effort compared to triggered NAVA.

## Background

Newborn infants with respiratory distress are prone to alveolar collapse and must compensate for a compliant chest wall (combined with stiff lungs) by maintaining end-expiratory lung volume above the relaxation volume. Maintenance of end expiratory lung volume (EELV) above the resting value involves a number of mechanisms including increased respiratory rate [1–5], constriction of the laryngeal muscles to brake expiratory flow [6], and the persistence of diaphragm electrical activity (EAdi) into expiration “EAdi_tonic_” [7]. With intubation, the upper airway constrictor muscles cannot contribute to this effect, putting greater “stress” on the diaphragm as a mechanism of maintaining an elevated EELV to help prevent lung de-recruitment.

The Hering-Breuer deflation sensitive reflex [8] was described more than 140 years ago, and describes a vagally-mediated process by which stretch receptors in the lung sense lung deflation. Lung deflation sends signals to the respiratory centers to stimulate the respiratory muscles which act to slow down exhalation. In intubated animals with acute lung injury (reduced compliance, edema, and atelectasis), removal of positive end expiration pressure (PEEP) causes an increased EAdi_tonic_ (with a concomitant loss of the cyclic “phasic EAdi”) [9, 10]. A gradual step-wise application of PEEP showed a dose–response reduction in tonic activity [9] and re-institution of phasic breathing. In theory, therefore, the “appropriate” amount of PEEP could be titrated by the magnitude of EAdi_tonic_.

In the present study, we introduce a new respiratory support device where the EAdi controls the delivery of assist continuously *both* during inspiration and during expiration, continuous Neurally Adjusted Ventilatory Assist (cNAVA). This is different from the conventional and commercially available mode known as Neurally Adjusted Ventilatory Assist (NAVA) which is triggered by the EAdi (tNAVA), but only provides assist in proportion to the inspiratory EAdi [11]. With cNAVA, the pressure delivered follows the EAdi waveform *continuously* during inspiration and expiration and should provide a neurally-adjusted expiratory pressure, as well as neurally-adjusted inspiratory assist.

We hypothesized that by adjusting pressure continuously (in proportion to both inspiratory and expiratory EAdi), cNAVA would not only offer inspiratory assist for tidal breathing, but also may aid in delivering a “neurally adjusted PEEP”, and more specific breath-by-breath unloading.

Both tNAVA and cNAVA were tested in animals undergoing different respiratory stresses, using both invasive and non-invasive ventilation. cNAVA was compared to tNAVA (without PEEP) in order to match the synchrony and proportionaIity obtained during inspiration.

Some of the results of this study previously been reported [12].

## Methods

The study was approved by St. Michael’s Hospital Animal Care and Use Committee. Care and handling of the animals were performed according to the Canadian Council on Animal Care.

### Animal instrumentation and measurements

Nine adult male New Zealand white rabbits (Charles River Labs, St. Constant, Quebec, Canada) with a mean body weight of 3.1 ± 0.2 kg were studied. For tNAVA, a Servo-i ventilator (Maquet, Sweden) was used (connected via tubing to the trachea (invasive) or to the nasal prongs (non-invasive). For cNAVA, a custom built respiratory support device was used (details given below).

Animal preparation has been previously described in detail [9, 13]. Briefly, animals received a continuous infusion of ketamine hydrochloride (10 mg/Kg/h), xylazine (2 mg/Kg/h) and lactated Ringer’s solution (5 mL/Kg/h). Arterial blood pressure (Pd 23, Gould Inc., Cleveland, OH) and blood for measurement of arterial blood gases (Ciba-Corning Model 248, Bayer, Leverkusen, Germany) were obtained from an ear artery. Transcutaneous oxygen saturation was measured with pulse oximetry (NONIN 8600 VTM, Nonin Medical Inc., Plymouth, MN) at the tail. Body temperature, measured with a rectal probe, was maintained between 38.5° and 39.5 °C with a heated surgical table.

For invasive ventilation, a tracheotomy was performed and an endotracheal tube (size 4.0) was inserted. For non-invasive delivery of assist, a double nasal prong (ID 3.5 mm) was inserted into the nostrils, and a t-piece replaced the endotracheal tube and was closed. EAdi was measured with an 8F oro-gastric catheter, with a balloon mounted for measurement of esophageal pressure (Pes). Proper positioning of the catheter was ascertained using a dedicated window on a Servo-i ventilator (“Catheter positioning window”). Esophageal balloon positioning was confirmed by the occlusion method [14]. Ventilator-delivered pressure (Pvent) and flow were obtained from a pneumotach (Novametrix Series 3 Neonatal flow sensor; Cat. No.:6718–00) placed between the tracheostomy tube (for invasive ventilation) or the nasal prongs (for non-invasive ventilation) and the y-piece of the ventilator’s circuit. At the end of the study procedure all animals were sacrificed by an overdose of anaesthesia.

### Method for tNAVA

**Fig. 1:**
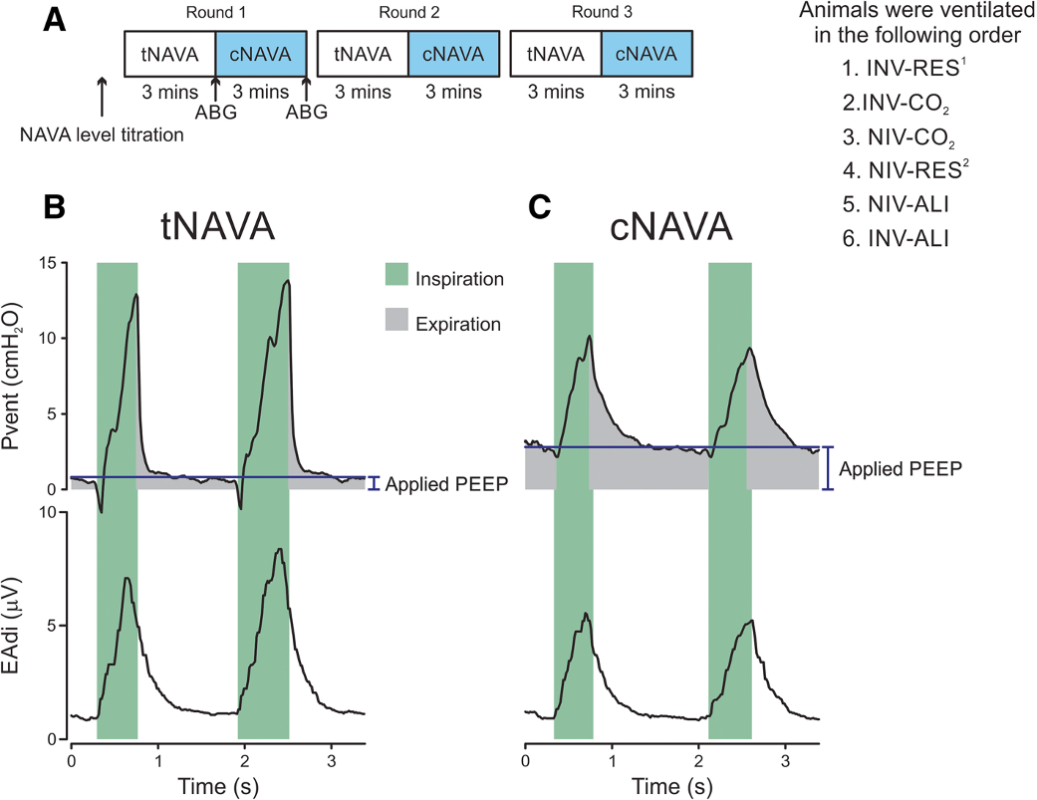
Schematic depiction of the order of procedures for each condition. Panel **a** For each condition, a tNAVA level titration was initially performed (see text for details). The same assist level was used for cNAVA. tNAVA and cNAVA were alternated, every 3 min, and repeated 3 times (3 “rounds”). Arterial blood gas samples were taken after 3 min of tNAVA, and after 3 min of cNAVA, only for round 1. Panel **b** and **c** Tracings of ventilator pressure (Pvent) and electrical activity of the diaphragm (EAdi) from one representative animal breathing on tNAVA (B) and cNAVA (C). Light green shading: neural inspiration; grey: pressure delivered during neural exhalation. Horizontal dashed line indicates expiratory pressure level (visual estimation) to demonstrate the higher pressure during cNAVA. INV-RES^1^ = Invasive ventilation with added resistive load; INV-CO_2_ = Invasive ventilation with CO_2_ blended in to air supply/medical air; INV-ALI = invasive ventilation with acute lung injury; NIV-RES^2^ = Non-invasive ventilation with added resistive load; NIV-CO_2_ = Non-invasive ventilation with CO_2_ blended in to air supply/medical air; NIV-ALI = Non-invasive ventilation after acute lung injury

tNAVA was delivered with the Servo-i ventilator. During tNAVA, the EAdi waveform was used to trigger on and cycle-off the ventilator, but also served to direct proportional assist on inspiration. Triggering occurred when the EAdi exceeded a threshold increment in EAdi (0.5 μV in the present study). After triggering, the assist was delivered in proportion to the EAdi ONLY throughout inspiration (the proportionality factor is the tNAVA level, in cm H_2_O per μV). The breath was cycled-off when the EAdi dropped to 70 % of peak, to a user-defined arbitrary PEEP (0 cmH_2_O in the present study) during neural expiration (Fig. 1b) [15].

### Method for cNAVA

Continuous NAVA was delivered with a custom-built ventilator. During cNAVA, pressure delivery (timing and amplitude) was controlled by the EAdi waveform, but *continuously* throughout the respiratory cycle (Fig. 1c). No triggering or cycling-off algorithms were used; the assist followed the EAdi waveform and can be considered a variable CPAP. Hence, both the maximum inspiratory pressure and end-expiratory pressure were controlled by the respiratory centers, along with the pressure produced by the ventilator in proportion to the EAdi signal. The magnitude of the assist (inspiratory and expiratory) was obtained by multiplying the EAdi by a gain factor- called the cNAVA level with the same units as the tNAVA level (cmH_2_O per μV. Instead of a fixed PEEP, cNAVA delivers “neurally adjusted-PEEP” according to EAdi_tonic_ (Fig. 1c).

### Protocol

The aim of the study was to compare cNAVA to tNAVA under different respiratory stresses. Figure 1a provides an overview of the Protocol. Animals were ventilated under six different and independent conditions, applied in the following non-randomized order:

1. Invasive ventilation with added resistive load (INV-RES^1^)
2. Invasive ventilation with CO_2_ blended in to air supply/medical air (INV-CO_2_)
3. Non-invasive ventilation with CO_2_ blended in to air supply/medical air (NIV-CO_2_)
4. Non-invasive ventilation with added resistive load (NIV-RES^2^)
5. Non-invasive ventilation after acute lung injury (NIV-ALI)
6. Invasive ventilation with acute lung injury (INV-ALI)

For each of the six conditions, tNAVA and cNAVA were alternated, every 3 min, and repeated 3 times.

Before each condition, the tNAVA level was set to zero and maintained for 1 min. Next, a tNAVA level titration procedure was performed to determine the optimal level of assist [16]. Briefly, the tNAVA level was increased by 0.2 cmH_2_O/μV every 10 breaths until a plateau was observed in Pvent (this was considered the adequate tNAVA level) [16]. During cNAVA, the same assist level was used. (Herein, we will simply refer to the gain factor as “NAVA level”).

No chin straps were used for the NIV portions of the study, and the stomach was not vented throughout these periods.

In each condition, at the end of the first 3 min paired comparison, an arterial blood gas sample was taken. The fraction of inspired oxygen (FiO_2_) was set to maintain oxygen saturation more than 90 %, and remained the same between modes in each condition.

#### Resistive load

For invasive ventilation, a custom-made resistive load was inserted between the endotracheal tube and the pneumotach (RES^1^). For non-invasive ventilation, a clamp between the nasal prongs and the y-piece of the ventilator’s circuit was used to increase resistance (RES^2^).

#### CO_2_ load

Inspiratory CO_2_ concentration was increased to 3.75–4.50 %, which resulted in an increase in end-tidal CO_2_ to 70–80 mmHg and a subsequent increase in central respiratory drive.

#### Acute lung injury (ALI)

Lung injury was induced by intratracheal instillation of 1.5 ml/kg of hydrochloric acid (pH 1.5) [10]. Arterial blood gas analysis was obtained at 5 min after HCl instillation and a PaO_2_/FiO_2_ lower than 150 was required to proceed to the following step; if this target was not reached, a second instillation of HCl (1 ml/kg) was performed [17].

### Data analysis

Off-line breath-by-breath analysis was performed on the acquired data for EAdi, Pes, flow, and Pvent waveforms for all rounds and all conditions. For both tNAVA and cNAVA, an average of the variables was calculated for the last minute of the three paired conditions.

Neural inspiratory time (Nti), neural expiratory time (Nte) and neural respiratory rate (Nrr) were determined from the EAdi signal. Tidal volume (Vt) was obtained by integrating the flow (for invasive conditions only). Peak, and mean inspiratory and expiratory values for Pvent, and EAdi were calculated. Inspiratory deflections in Pes were calculated between the onset and the negative peak. The predicted (i.e. mathematically calculated) PEEP during both modes was calculated by mean expiratory EAdi × cNAVA level, and this was compared to the measured mean expiratory pressure. Neuro-ventilatory efficiency (NVE) was calculated as Vt (ml)/delta inspiratory change in EAdi (μV) [18–20].

### Statistical analysis

Statistical analysis was performed with SigmaPlot software package (v 12, Systat Software). Results are presented as mean ± SD, unless otherwise indicated. Two-way Repeated Measures ANOVA (with Two Factor Repetition) was used to compare the impact of Mode (cNAVA vs. tNAVA) during all rounds and for all six conditions. Pairwise Multiple Comparisons were performed with the Student-Newman-Keuls method. A significant difference was defined as *P* < 0.05. Bland-Altman plots were used to compare the relationship between mathematically predicted expiratory pressure and actual applied expiratory pressure, in both modes [21].

## Results

The NAVA levels that were used in each condition are shown in Table 1.

**Table 1:** NAVA level and respiratory variables during tNAVA and cNAVA for each condition (*n* = 9)

		Invasive			Non-invasive		
		RES^1^	CO2	ALI	RES^2^	CO2	ALI
NAVA level (cm H2O/μV)		5.5 ± 0.4	2.1 ± 0.2	2.2 ± 0.6	4.1 ± 0.8	2.6 ± 0.4	2.6 ± 0.5
Nti (sec)	tNAVA	0.67 ± 0.06	0.50 ± 0.06	0.47 ± 0.09	0.71 ± 0.11	0.63 ± 0.10	0.56 ± 0.11
	cNAVA	0.62 ± 0.04*	0.47 ± 0.06*	0.45 ± 0.07*	0.71 ± 0.15	0.60 ± 0.08*	0.54 ± 0.10
Nte (sec)	tNAVA	1.41 ± 0.24	0.93 ± 0.21	1.11 ± 0.54	1.67 ± 0.52	1.16 ± 0.34	0.95 ± 0.48
	cNAVA	1.56 ± 0.30*	0.99 ± 0.24	1.10 ± 0.53	1.87 ± 0.54*	1.28 ± 0.37*	1.06 ± 0.49
Nti/Tot (%)	tNAVA	0.33 ± 0.03	0.36 ± 0.05	0.34 ± 0.11	0.32 ± 0.07	0.36 ± 0.07	0.41 ± 0.09
	cNAVA	0.29 ± 0.03*	0.33 ± 0.04*	0.32 ± 0.09	0.29 ± 0.05*	0.33 ± 0.07*	0.36 ± 0.07*
Nrr (per min)	tNAVA	30.2 ± 3.9	43.9 ± 7.5	46.9 ± 27.5	28.4 ± 10.1	35.8 ± 8.3	47.7 ± 21.9
	cNAVA	28.8 ± 4.4	42.9 ± 7.0	46.0 ± 21.5	26.1 ± 9.0	33.8 ± 7.2	42.8 ± 15.5

Data are presented as average value of the means and standard deviation all 3 round s in each condition. *p < 0.05 cNAVA compared to tNAVA in the same condition

*INV-RES*^*1*^ Invasive ventilation with added resistive load, *INV-CO*_*2*_ Invasive ventilation with CO_2_ blended in to air supply/medical air, *INV-ALI* invasive ventilation with acute lung injury, *NIV-RES*^*2*^ Non-invasive ventilation with added resistive load, NIV-CO_2_ Non-invasive ventilation with CO_2_ blended in to air supply/medical air, *NIV-ALI* Non-invasive ventilation after acute lung injury, *Nti* Neural inspiratory time, *Nte* Neural expiratory time, *Nti/Tot* Neural inspiratory duty cycle, *Nrr* Neural respiratory rate

**Fig. 2:**
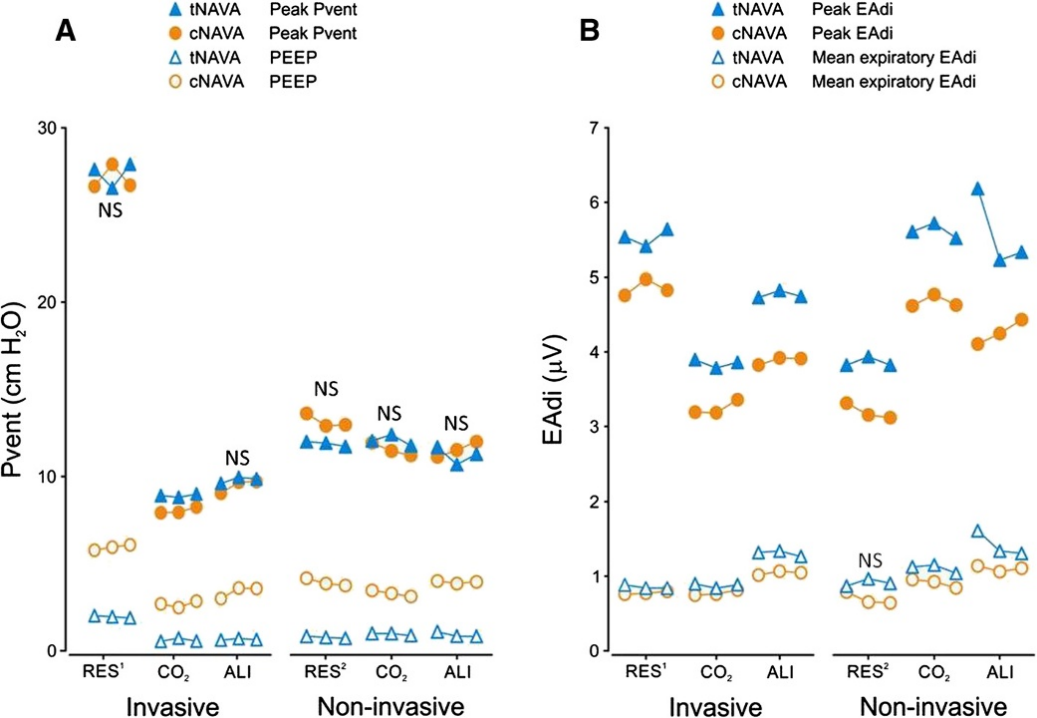
Ventilator-delivered pressure and EAdi during triggered NAVA and continuous NAVA for all rounds, during all six conditions. Panel **a** Ventilator pressure (Pvent) values (Y axis) (peak Pvent = solid symbols; mean expiratory Pvent = open symbols) are plotted for the six conditions (X axis) for tNAVA (blue) and cNAVA (orange). Despite similar peak pressure, cNAVA consistently delivered higher mean expiratory pressure, and hence, lower inspiratory (delta Pvent) assist compared to tNAVA. (Note: during tNAVA, PEEP is manually set to zero as described in the Protocol section). Panel **b** Diaphragm electrical activity (EAdi) (Y axis) (peak EAdi = solid symbols; mean expiratory EAdi = open symbols) are plotted for the six conditions (X axis) for tNAVA (blue) and cNAVA (orange). For both Panels A and B, values plotted are the mean of the last minute of each run. Only non-significance between modes is indicated (NS) for a given condition. INV-RES^1^ = Invasive ventilation with added resistive load; INV-CO_2_ = Invasive ventilation with CO_2_ blended in to air supply/medical air; INV-ALI = invasive ventilation with acute lung injury; NIV-RES^2^ = Non-invasive ventilation with added resistive load; NIV-CO_2_ = Non-invasive ventilation with CO_2_ blended in to air supply/medical air; NIV-ALI = Non-invasive ventilation after acute lung injury; NS = No significant difference tNAVA compared with cNAVA within the same condition

Figure 2 demonstrates for all six conditions, the ventilator pressures (panel A) and the EAdi variables (panel B) for each of the three rounds. Peak Pvent was not different for tNAVA and cNAVA, in all conditions, except for one (INV-CO_2_). During INV-CO_2_ peak Pvent was significantly lower in cNAVA than in tNAVA, but by less than 1 cm H_2_O (8.2 ± 0.2 vs. 9.0 ± 0.1 cmH_2_O), for the mean of the three rounds.

The distribution of inspiratory and expiratory pressures was different between tNAVA and cNAVA. For each condition, the mean expiratory Pvent was always significantly higher during cNAVA than tNAVA. During cNAVA, the mean expiratory Pvent for all conditions was 4.0 ± 1.1 cmH_2_O, and significantly higher than value during tNAVA (1.1 ± 0.2 cmH_2_O) (*P* <0.05) for each condition. By default, mean inspiratory Pvent (Pk Pvent – mean expiratory Pvent) was lower during all conditions in cNAVA compared to tNAVA. Relative to tNAVA, the mean inspiratory Pvent during cNAVA was reduced on average (for all conditions) by 40 % (range 24 %–51 %).

Peak EAdi was always significantly lower in cNAVA than in tNAVA (Fig. 2, Panel b). The mean expiratory EAdi was also lower during cNAVA compared with tNAVA, in five of the six conditions (INV-RES^1^, INV-CO_2_, INV-ALI, NIV-CO_2_ and NIV-ALI respectively, *P* < 0.05), and tended to be lower during NIV-RES^2^. Compared with tNAVA, mean inspiratory EAdi was therefore lower in cNAVA during all the six conditions. Relative to tNAVA, mean inspiratory EAdi was reduced on average (for all conditions) by 19 % (range 14 %–25 %).

**Fig. 3:**
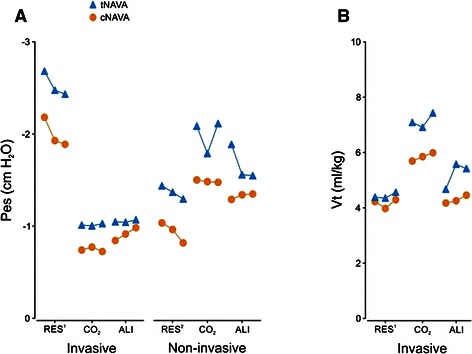
Esophageal pressure (Pes) and tidal volume during triggered NAVA and continuous NAVA for all rounds, during different conditions. Panel **a** Esophageal pressure swing (Y axis) plotted for the six conditions (X axis) for tNAVA (blue) and cNAVA (orange). For each condition, the esophageal pressure swing was less for cNAVA than tNAVA, indicating less inspiratory effort. (Note, the more negative the value, the greater the inspiratory effort). Panel **b** Inspired tidal volume (Y axis) plotted for the invasive conditions (X axis) for tNAVA (blue) and cNAVA (orange). For both Panels A and B, values plotted are the mean of the last minute of each run. All comparisons between cNAVA and tNAVA, within a condition, were significantly different between the 2 modes. INV-RES^1^ = Invasive ventilation with added resistive load; INV-CO_2_ = Invasive ventilation with CO_2_ blended in to air supply/medical air; INV-ALI = invasive ventilation with acute lung injury; NIV-RES^2^ = Non-invasive ventilation with added resistive load; NIV-CO_2_ = Non-invasive ventilation with CO_2_ blended in to air supply/medical air; NIV-ALI = Non-invasive ventilation after acute lung injury

In agreement with the EAdi data, cNAVA significantly decreased the inspiratory swing in Pes (Fig. 3, Panel a). On average, the inspiratory Pes deflection was reduced by 22 % during cNAVA for the six conditions (range 18 %–28 %) compared with tNAVA, *p* < 0.05.

The ventilatory pattern in cNAVA and tNAVA is reported in Table 1. Neural inspiratory time (Nti) tended to be lower in cNAVA compared with tNAVA, and reached statistical significance during four of the six conditions (*P* < 0.05 for INV-RES^1^, INV-CO_2_, INV-ALI, and NIV-CO_2_). During cNAVA, neural expiratory time (Nte) was significantly longer in three conditions (INV-RES^1^, NIV-RES^2^, NIV-CO2), and tended to be longer in the other three. Neural Ti/Ttot was significantly lower in cNAVA than tNAVA during all conditions, but one (*P* < 0.05 INV-ALI). Neural respiratory rate was always similar between the two modes for all conditions.

Vt was significantly lower in cNAVA than tNAVA in all the invasive conditions (*p* < 0.05) (Fig. 3, Panel b). Vt could not be reliably measured with the leak in NIV conditions. Neuroventilatory efficiency, calculated as Vt/EAdi, was comparable for all invasive conditions.

**Fig. 4:**
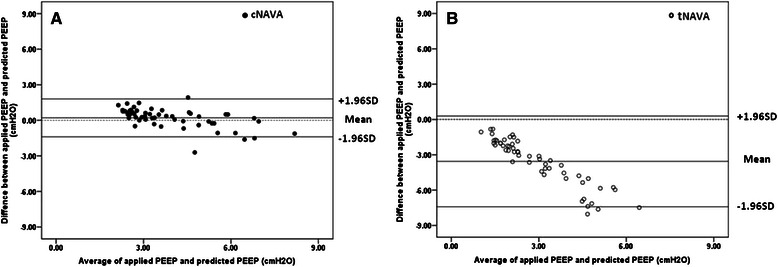
Limits of agreement between predicted PEEP and applied PEEP. Horizontal dashed line indicates zero difference. Solid horizontal lines indicate the mean and 95 % confidence intervals. Panel **a** Bland-Altman plot for all conditions during cNAVA (solid symbols) shows very little difference between mathematically predicted PEEP and applied PEEP. The mean difference was 0.2 ± 0.8 cm H_2_O. Panel **b** Bland-Altman plot for all conditions during tNAVA (open symbols) shows a greater little difference between predicted and applied PEEP. The mean difference was −3.6 ± 2.0 cm H_2_O

Figure 4a (cNAVA) and B (tNAVA) demonstrate Bland-Altman plots for the “mathematically predicted PEEP” (mean expiratory EAdi × NAVA level) vs. the actual applied PEEP (Mean expiratory Pvent), for all conditions. During cNAVA, the mean difference between the predicted PEEP and the applied PEEP was 0.2 ± 0.8 cm H_2_O. For tNAVA, the mean difference was −3.6 ± 2.0 cm H_2_0, and the applied PEEP was always lower than the predicted PEEP.

Mean arterial blood pressure was lower during cNAVA compared to tNAVA in all conditions (*p* < 0.05, Table 2). In general, arterial blood gas values were not different between modes. However, there were two exceptions: PaO_2_ significantly increased when animals were switched from tNAVA to cNAVA during invasive ventilation after ALI (*P* < 0.05), and was associated with an increase in PaO_2_ /FiO_2_, which did not reach statistical significance (*P* = 0.067) (Table 2). In INV-ALI, pH was also higher during cNAVA compared with tNAVA (*P* < 0.05).

**Table 2:** Arterial blood gas values and blood pressure during tNAVA and cNAVA for each condition (*n* = 9)

		Invasive			Non-invasive		
		RES^1^	CO2	ALI	RES^2^	CO2	ALI
pH	tNAVA	7.34 ± 0.03	7.34 ± 0.04	7.29 ± 0.06	7.32 ± 0.02	7.33 ± 0.04	7.33 ± 0.06
	cNAVA	7.34 ± 0.03	7.33 ± 0.05	7.28 ± 0.06*	7.32 ± 0.02	7.33 ± 0.04	7.31 ± 0.06
PaO_2_ (mm Hg)	tNAVA	192 ± 114	131 ± 25	92 ± 32	165 ± 113	176 ± 45	71 ± 21
	cNAVA	195 ± 113	143 ± 40	119 ± 55*	174 ± 124	180 ± 32	78 ± 27
FiO_2_ (%)	tNAVA	41 ± 4	38 ± 9	90 ± 10	43 ± 29	46 ± 8	87 ± 8
	cNAVA	41 ± 4	38 ± 9	90 ± 10	43 ± 29	46 ± 8	87 ± 8
PaO_2_/FIO_2_	tNAVA	479 ± 53	315 ± 42	107 ± 54	400 ± 113	385 ± 70	83 ± 32
	cNAVA	490 ± 50	344 ± 83	140 ± 90	417 ± 125	397 ± 54	91 ± 38
PaCO_2_ (mm Hg)	tNAVA	64 ± 3	61 ± 3	52 ± 11	66 ± 3	58 ± 11	52 ± 13
	cNAVA	64 ± 6	62 ± 7	53 ± 15	66 ± 4	57 ± 13	52 ± 11
SaO_2_ (%)	tNAVA	98 ± 2	98 ± 1	93 ± 4	97 ± 2	99 ± 0	91 ± 4
	cNAVA	98 ± 2	98 ± 1	97 ± 3	94 ± 9	99 ± 0	92 ± 4
MAP (mm Hg)	tNAVA	82 ± 6	81 ± 8	65 ± 10	74 ± 12	70 ± 12	66 ± 12
	cNAVA	80 ± 8*	76 ± 6*	59 ± 11*	72 ± 10*	67 ± 13*	64 ± 12*

Data are presented mean and standard deviation for all animals after the first round, in each condition. *p < 0.05 cNAVA compared to tNAVA in the same condition

*INV-RES*^*1*^ Invasive ventilation with added resistive load, *INV-CO*_*2*_ Invasive ventilation with CO_2_ blended in to air supply/medical air, *INV-ALI* invasive ventilation with acute lung injury, *NIV-RES*^*2*^ Non-invasive ventilation with added resistive load, *NIV-CO*_*2*_ Non-invasive ventilation with CO_2_ blended in to air supply/medical air, *NIV-ALI* Non-invasive ventilation after acute lung injury, *PaO*_*2*_ oxygen tension in arterial blood, FiO_2_ fraction of inspired oxygen, *PaCO*_*2*_ carbon dioxide tension in arterial blood, *SaO*_*2*_ Arterial oxygen saturation, *MAP* mean arterial blood pressure

## Discussion

### Summary of the results

This is the first study to show that during both invasive and non-invasive ventilation, and during different conditions of respiratory stress, cNAVA is capable of delivering proportional assist both on inspiration (neurally adjusted inspiratory pressure) and expiration (neurally adjusted PEEP). During cNAVA, the mean expiratory pressure was higher, and the mean inspiratory pressure was lower, than during tNAVA, for similar peak pressures. This was associated with less inspiratory effort (indicated by lower esophageal pressure swings and lower inspiratory EAdi) and lower mean expiratory diaphragm activity during cNAVA compared to tNAVA. These results, taken all together indicate that cNAVA was more efficient at ventilation and unloading than tNAVA (i.e. less ventilator-pressure was required for unloading).

### Clinical relevance

It has been demonstrated that neural breathing pattern in infants is more complex than previously believed [22], where both phasic EAdi and tonic EAdi are extremely variable (~90 % variability), and where tonic EAdi is quite prevalent (30 % of breathing is spent with elevated tonic EAdi). This suggests that applying a fixed level of assist and a fixed level of PEEP during mechanical ventilation, or even a fixed CPAP during NIV may not be suitable. For example, Emeriaud et al. showed in mechanically ventilated infants, that tonic activity of the diaphragm was 12 % of the inspiratory EAdi at prescribed PEEP levels, which suggested insufficient PEEP application [23].

The best method of selecting the optimal PEEP level is still controversial. cNAVA has potential implications in relation to setting individualized, real time PEEP levels in infants and ARDS adults who have tonic diaphragm activation due to lung collapse. cNAVA is a method of applying PEEP on a breath-by-breath basis in a manner that is “controlled” by the patient’s underlying physiologic integration of multiple inputs. We understand that whether this approach would be beneficial for the patient is uncertain at the moment, and may vary depending on the disease process.

In addition, the growing use of NIPPV in preterm neonates [24] demands a synchronized, proportional, and variable mode, which is not affected by leaks [25]. cNAVA could offer the opportunity of delivering assist, which in essence is a true mix of CPAP and NIPPV, without triggering, and which neurally adjusts both the inspiratory and expiratory pressures.

### Physiology of Tonic EAdi and PEEP

Hering-Breuer initially described the “deflation-sensitive” reflex in 1868, where cats showed increased inspiratory activity with reductions in relaxation volume [26]. About one hundred years later, Luck [27] demonstrated discharging afferent vagal fibers during expiration, when negative intratracheal pressure was applied in spontaneously breathing (yet anesthetized rabbits). Allo [9] and Beck [10], both showed after ALI induced by HCl (lung collapse, altered lung compliance, edema) that diaphragm electrical activity was increased during the exhalation period (also known as “tonic EAdi”, in rabbits who were intubated without PEEP (upper airways could not participate in EELV maintenance). This tonic EAdi is abolished by vagotomy [9]. Other factors shown experimentally to increase tonic EAdi include abdominal distension [28], and application of continuous negative pressure [29]. In adult humans, lung deflation with continuous negative airway pressure is associated with tonic diaphragm activity [30]. Tonic EAdi has also been described and quantified in non-ventilated premature infants [22]: measured over several days, these infants were found to spend one third of their time breathing with elevated tonic activity. Tonic EAdi was even observed in mechanically ventilated infants who were being ventilated with PEEP [23].

Is it disadvantageous to have increased tonic EAdi? A maintained and increased tonic activity could have a negative impact on respiratory efficiency via several mechanisms, including inadequate “rest” between breath cycles, decreased diaphragm blood circulation (because of higher diaphragm tension), and increased diaphragm metabolism, as suggested by Emeriaud [23]. Application of PEEP reduces tonic EAdi [9, 10, 23]. Incremental PEEP application was associated with a concomitant decrease in tonic EAdi, which was reversible [9]. Increasing PEEP recruits the lung and increases end-expiratory lung volume [31–33]. One interpretation of these data was that the tonic EAdi was indicative of the level of EELV that was physiologically necessary for that particular breath. This concept led to the development of “Continuous NAVA” (cNAVA). In the present study, cNAVA was shown to be feasible, and to deliver neurally adjusted expiratory pressure under different respiratory conditions, in anesthetized and spontaneously breathing rabbits. In healthy volunteers, a prototype version of cNAVA also demonstrated the ability to deliver neurally adjusted PEEP, albeit in a single subject [15].

### Impact of cNAVA on unloading

The consequence of delivering proportional assist on both inspiration and expiration with cNAVA, was a reduced swing in esophageal pressure, and lower mean inspiratory diaphragm activity, both indicating reduced inspiratory effort (unloading). This reduced effort occurred despite the lower inspiratory assist during cNAVA.

Application of PEEP during intubation and mechanical ventilation can reduce work of breathing [34–36], and studies have mainly focused on the work of breathing in relation to triggering during conventional “pneumatically-triggered” modes. In the present study, we used neurally controlled modes (tNAVA and cNAVA), and the work of breathing (and diaphragm activation) due to triggering is minimized in both modes [37, 38]. Since there is no triggering of an inspiratory valve during cNAVA, one could speculate that the reduced inspiratory effort is due to “no triggering required”, compared to tNAVA. Perhaps the increased PEEP in itself reduced inspiratory effort by any of the following (or combination thereof): improved respiratory system mechanics, improved chest wall configuration, diaphragm at a more advantageous portion of the length-tension curve, although these were not measured in the present study.

Passath et al. [18] have demonstrated in a mixed group of intubated and ventilated patients that the response to increasing PEEP is a reduced EAdi. They examined “neuro-ventilatory efficiency, NVE” [18–20] during PEEP titrations while ventilated with NAVA, and identified individualized PEEP levels, where tidal breathing could occur with a minimal inspiratory EAdi. In the present study, NVE could only be measured during invasive conditions and was not affected by mode, probably because the increased end-expiratory pressure during cNAVA led to a simultaneous decline of EAdi and Vt which resulted in the constant ratio of Vt/EAdi.

Another factor influencing respiratory drive is arterial blood gases. PaCO_2_ was the same during cNAVA and tNAVA, for all six conditions. Considering that acute hypoxia can increase respiratory drive [39], we speculate that the significant improvement in PaO2 with cNAVA may have reduced the inspiratory EAdi.

### Potential limitations of cNAVA

For any neurally controlled mode of ventilation, there are limitations. Obviously, the requirement for these modes to function and be safe is the presence of an EAdi signal, with a cautious use in patients with uncontrollable respiratory drive. Similar to tNAVA, cNAVA requires upper pressure limits and backup ventilation and alarms for safety. In addition, even if phasic EAdi is present, the absence of tonic EAdi would require a lower pressure (PEEP) limit (guarantee), which can be set by the user.

In the event of high tonic Edi, cNAVA may provide too high levels of expiratory pressure, possibly compromising hemodynamics [40] or causing apnea [9] However, in the present study, we did not observe such high end-expiratory pressures (highest 6 cm H_2_O), and apnea was never observed. We did find a statistically lower mean arterial blood pressure during cNAVA, but it is difficult to interpret the clinical implications of this drop (and was on average only 3.3 mmHg lower). In the future, a safety limit could be applied to the end-expiratory pressure to ensure it does not exceed a compromising level.

### Limitations of study

The aim of this study was to compare cNAVA to tNAVA under different respiratory conditions that would increase respiratory drive (both EAdi phasic and EAdi tonic). However, the study was not designed to compare the actual conditions (e.g. INV-ALI vs. NIV-ALI). Therefore, our statistical test of choice was a two-way RM ANOVA within a given condition (and not between conditions), which allowed us to also test reproducibility. Along the same topic, the resistive loads during both resistance conditions (INV-RES^1^ and NIV-RES^2^) were different, and not quantified. However, the resistance was always the same when comparing cNAVA and tNAVA within a condition (and was the same for all animals). The order of the conditions tested was not randomized. Obviously, the ALI model is non-reversible, and needed to be performed as the last conditions, and could not be randomized.

In order to determine the adequate level of assist, we used a tNAVA level titration procedure as described by Brander [16], but using the tNAVA mode, which delivers proportional assist on inspiration only. We did not repeat the titration with cNAVA, and therefore, we can only assume that the adequate level “selected” by the subject was appropriate for neurally adjusting the PEEP as well. Future studies will help to determine the “best” way of setting the cNAVA level.

In the present study, the PEEP during tNAVA was fixed and standardized to 0 cm H_2_O. This provided us with a condition of high tonic Edi and largest Pes swings, and thus the effect of cNAVA could be compared to those. We wanted to show that it would be feasible to deliver expiratory pressure during exhalation, and that this would lead to reduced Edi and Pes swings. Future studies could be performed using different levels of fixed PEEP during tNAVA.

Our ventilation periods for each condition and mode (3 min) could be criticized as being short, however, the aim of this study was to test the capability of cNAVA in delivering neurally adjusted PEEP, and the repeated cross-over design allowed the elimination of carry-over effects. Longer application of cNAVA would of course be required to evaluate for more important effects.

## Conclusion

We demonstrated that Continuous NAVA is feasible and delivers “neurally-adjusted PEEP” in proportion to EAdi during expiration. Compared to triggered NAVA, cNAVA decreased both respiratory drive and inspiratory effort.

## Abbreviations

ALI: Acute lung injury
cNAVA: Continuous Neurally Adjusted Ventilatory Assist
CPAP: Continuous Positive Airway Pressure
EAdi: Diaphragm electrical activity
EAdi_tonic_: diaphragm electrical activity during expiration
EELV: end expiratory lung volume
INV-ALI: Invasive ventilation with acute lung injury
INV-CO_2_: Invasive ventilation with CO_2_ blended in to air supply/medical air
INV-RES: Invasive ventilation with added resistive load
NAVA: Neurally Adjusted Ventilatory Assist
NIV-CO_2_: Non-invasive ventilation with CO_2_ blended in to air supply/medical air
NIV-RES: Non-invasive ventilation with added resistive load
NIV-ALI: Non-invasive ventilation after acute lung injury
nRR: Neural respiratory rate
nTi: Neural inspiratory time
nTe: Neural expiratory time
NVE: Neuro-ventilatory efficiency
PEEP: positive end-expiratory pressure
Pes: esophageal pressure
Pvent: Ventilator-delivered pressure
tNAVA: triggered and cycled-off Neurally Adjusted Ventilatory Assist
Vt: Tidal volume
